# Automatic Actionable Information Processing and Trust Management towards Safer Internet of Things

**DOI:** 10.3390/s21134359

**Published:** 2021-06-25

**Authors:** Marek Janiszewski, Anna Felkner, Piotr Lewandowski, Marcin Rytel, Hubert Romanowski

**Affiliations:** Research and Academic Computer Network (NASK), Kolska 12, 01-045 Warsaw, Poland; anna.felkner@nask.pl (A.F.); piotr.lewandowski@nask.pl (P.L.); marcin.rytel@nask.pl (M.R.); hubert.romanowski@nask.pl (H.R.)

**Keywords:** Internet of Things (IoT), vulnerabilities, vulnerability database, exploits, TRM, trust, trust and reputation management

## Abstract

The security of the Internet of Things (IoT) is a very important aspect of everyday life for people and industries, as well as hospitals, military, households and cities. Unfortunately, this topic is still too little researched and developed, which results in exposing users of Internet of Things to possible threats. One of the areas which should be addressed is the creation of a database of information about vulnerabilities and exploits in the Internet of Things; therefore, the goal of our activities under the VARIoT (Vulnerability and Attack Repository for IoT) project is to develop such a database and make it publicly available. The article presents the results of our research aimed at building this database, i.e., how the information about vulnerabilities is obtained, standardized, aggregated and correlated as well as the way of enhancing and selecting IoT related data. We have obtained and proved that existing databases provide various scopes of information and because of that a single and most comprehensive source of information does not exist. In addition, various sources present information about a vulnerability at different times—some of them are faster than others, and the differences in publication dates are significant. The results of our research show that aggregation of information from various sources can be very beneficial and has potential to enhance actionable value of information. We have also shown that introducing more sophisticated concepts, such as trust management and metainformation extraction based on artificial intelligence, could ensure a higher level of completeness of information as well as evaluate the usefulness and reliability of data.

## 1. Introduction

According to the Cambridge Online Dictionary [1], “Internet of Things” refers to the “objects with computing devices in them that are able to connect to each other and exchange data using the Internet”. Therefore, virtually any system that consists of interconnected computing devices that have unique identifiers and can transfer data over a network without human or computer interaction is an example of the “Internet of Things”. However, the world of IoT is evolving and the above definition is not the only accepted definition of an IoT device. For this reason, for the purposes of our research, we adopted the following definition: “IoT device—an item (except a phone, PC, tablet and data center hardware) equipped with network connectivity and the ability to collect and exchange data”. Not only is the scale of use of these devices growing but so is their importance. They are used not only at home but also in hospitals, the military and industry, therefore ensuring the security of Internet of Things devices is more and more urgent and necessary.

According to IoT Analytics [2], for the first time in 2020 there were more IoT devices (e.g., connected cars, smart home devices, connected industrial devices) than non-IoT devices (smartphones, laptops and computers), and it is estimated that by 2025 there will be over 30 billion IoT devices, on average almost four IoT devices per person. Therefore, ensuring the cybersecurity of these devices is essential from the point of view of each stakeholder.

The security of the Internet of Things is clearly lacking, as evidenced by the large-scale incidents such as Mirai-like botnets. IoT security is therefore a major focus of research plans at many levels, including in the “Input to the Horizon Europe Programme 2021–2027 Priorities for the definition of a Strategic Research and Innovation Agenda in Cybersecurity” [3]. National strategies are even clearer on this point. The “Cybersecurity Research Analysis Report for Europe and Japan” [4] shows that the Internet of Things is one of the most commonly addressed research areas in the European national cybersecurity strategies, as well as being one of the common interests between the EU and Japan, showing that the issue is vital on a global scale.

The IoT cybersecurity data landscape is extremely fragmented, with different data formats and models, gaps in available information, significant data quality issues and a lack of global research. This makes the cost of obtaining such data for interested entities high, as even the identification of the most useful sources is an important and difficult task.

The multitude of existing vulnerabilities, different vendor responses to them and weak or nonexistent patching processes pose a serious threat to both the security of citizens and the economy. Infected IoT home appliance devices can be used to steal users data, spy on them or lead to damages (e.g., fire, flood or burglary). Infected industrial IoT devices can be used to disrupt technological processes where they are involved or cause damages. All sorts of infected IoT devices can be used for distributed attacks on other digital services and assets. Solving these problems is much more difficult due to the lack of rich common sources of actionable information about IoT vulnerabilities, known exploits and incidents recorded in the wild. Such services are necessary to support the proper response of vendors, service providers, mitigation activities of network owners, development of services increasing the security of end users as well as further research activities in the field of cybersecurity in the IoT world.

Having information about vulnerabilities in one’s devices is extremely important from the point of view of producers, service providers, network owners and device owners. Obtaining such information is also crucial from the point of view of national and sectoral CSIRTs (Computer Security Incident Response Team). Vulnerability management is an extremely important aspect of security both in the IT and IoT world. Vulnerability management can also be used to determine risk assessment at various levels, as it has been presented, for example, in the article [5].

The paper is organized as follows. Section 2 provides a broad overview of our system. Section 3 discusses data sources and methods of obtaining information from them. Methods of aggregating collected data are presented in Section 4. Data filtering mechanisms, needed to select IoT related entries are discussed in Section 5. Section 6 presents an AI-supported approach of extracting metadata from raw text entries. It is followed by Section 7, which discusses evaluation of trust to the data sources. Finally, results achieved by our system are shown in Section 8, which is followed by a summary and plans for future works in Section 9.

## 2. General Aim and Approach

The lack of a repository aggregating information about vulnerabilities and exploits of IoT devices, which could provide a high level of maturity, is a worrying problem currently; therefore, creation of such repository is the main focus of the article. We intend for it to include vulnerabilities and exploits related to hardware, firmware as well as software (if applicable) of IoT devices. Creation of the repository should take into account its usefulness and ability to process information in an automatic way. One of the most important assumptions is the need of harvesting information from many distinctive sources and combining them in consistent and unified entities, to enable access and use in various applications. One of such applications is vulnerability management of owned devices and systems built on the basis of these devices. Another not obvious application is the analysis and monitoring of the quality (in the context of cybersecurity) of vendors or their products, which may allow predicting the existence of new unknown vulnerabilities. Such a concept, although not dedicated to the IoT world, can be found in the article [6]. From the perspective of IoT devices, such prospects can be even more important and more promising.

**Figure 1:**
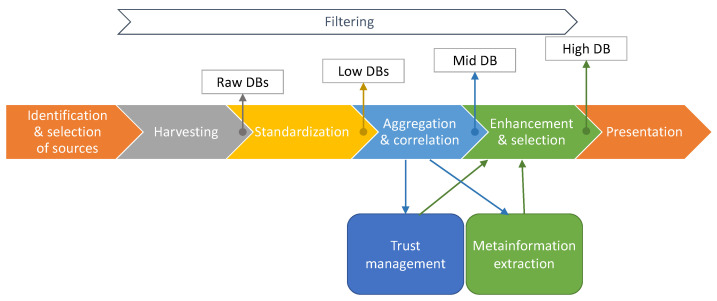
Stages and mechanisms to process information and outputs.

The process of creating the intended repository of vulnerabilities and exploits is shown in Figure 1 and can be briefly described as follows. The first step is the identification and selection of valuable sources of information related to vulnerabilities and exploits. Many types of sources, such as national vulnerability databases, CSIRTs and vendor’s bulletins and other structured sources are interesting. It is worth mentioning that unstructured sources, such as blogs, reports or individual websites can also be included in the repository. The next step consists of harvesting information from the sources and saving them in the so-called *raw databases*. In the next step, the information is standardized—for example, the names of the corresponding fields are unified and some supplementary information is added. As a result, the so-called *low databases* are created. These three first steps are described in Section 3. The aim of the fourth step is to correlate and then aggregate information from various sources about a vulnerability or an exploit. Details about that process can be found in Section 4. On the output of that process, the *medium database* is created. The *medium database* contains all the information from all *low databases* and every entry in that database corresponds to one vulnerability or exploit. Every field within an entry contains information derived from corresponding fields from *low databases*. The next step is to try to enhance and select the most reliable information about every vulnerability and exploit. More details about this process can be found in Section 5. This process uses two separate mechanisms, such as metainformation extraction and trust management, described in Section 6 and Section 7, respectively. Creation of *high database* is an output of this process. *High database* can be then shared and used for various purposes. To facilitate this, the last step—presentation—should be done.

Filtering at different levels is also done to select information related to IoT. Various means are used to complete this task, such as: internal IoT devices catalogue, self-created taxonomy for IoT devices, filtering mechanism based on keywords and, to some extent, information about devices from sources of information about vulnerabilities and exploits (in the minority, as not many of them provide useful information in this context). However, the filtering process is not trivial; it will not be described in detail in this article.

**Figure 2:**
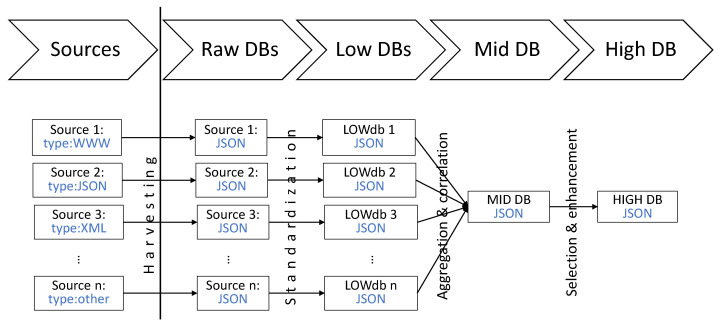
Architecture of DB and stages of processing information.

Figure 2 presents the architecture of the repository from the perspective of types of databases which are used to store information on various steps of processing. As it has been mentioned earlier, many types of sources can be useful for the purpose of this work. The information contained in these sources can be provided in various formats. What is even more interesting is that various mechanisms should be used to harvest information from the sources to create a *raw database* for each corresponding source. In the *raw database* there is no interference with the structure or the content of the information, but the information is saved in a common format—as a JSON file. Each *raw database* consists of information harvested from one source. Of course, every *raw database* can have many entries and contain information about many vulnerabilities or exploits. After standardization process, the *low databases* are created.

The number of the *low databases* is equal to that of *raw databases*; however, the number of entries and the structure of data differ due to standardization process. The *medium database* is created by combining information from all *low databases*. All information about a vulnerability or an exploit is combined into a single entry. Various mechanisms (such as identifiers matching or other means of correlation) are used to identify which entries in *low databases* correspond to the same vulnerability or exploit. It is worth mentioning that any vulnerability or exploit can be described in many sources, so on the level of *raw* and *low databases* as well as *medium database*, the information can be multiplicated. The last instance of the repository, the *high database*, is done to present comprehensive information about any vulnerability or exploit by selecting the most reliable piece of information in each parameter or by enhancement of existing information.

The architecture of the repository enables easily incorporating new information sources in the final information. Adding a new source of information needs three actions:

1. Harvesting information (by downloading or scraping);
2. Transforming information to the common format (creating a *low database* for that source);
3. Setting the trust value of the new source (or implement a more sophisticated trust calculation algorithm for that source; currently this is not and probably never will be needed, but our architecture supports inclusion of such mechanism).

Creation of *medium* and *high database* with use of the newly added source will be done automatically.

On the technical layer, various technologies are used to harvest and process information, such as: Python scripts with various libraries (at all steps), Selenium (to harvest information from selected sources), Elasticsearch and Kibana (as a database and to process and analyze information).

## 3. Information Harvesting and Standardization

We are obtaining data from multiple sources with varying data formats and different languages. The broad description of vulnerability information sources was published in [7]. In addition to the vulnerabilities, we are also obtaining exploit data from Packet Storm [8] and Exploit-DB [9]. All structured data sources currently in use are listed in Table 1.

Only publicly available free sources are considered, which disqualifies paid services such as vulnerability and exploit aggregator Vulners [21]. Besides the structured sources listed in Table 1, we are also obtaining write-ups, such as blogposts, about IoT vulnerabilities and exploits. In their case relevant metadata can be extracted from raw text, as discussed in Section 6.

From the data acquisition perspective, the sources can be divided into three categories:

1. Sources with API access;
2. Sources sharing data feeds;
3. Sources offering only a website.

Sources with API access are a minority among publicly available free sources. Out of sources listed in Table 1, an API is available only in JVNDB and, since March 2021, NVD [22]. There are more sources, usually national vulnerability databases, that offer data feeds in various formats: as JSON files (NVD), XML files (JVNDB, CNNVD and CNVD) or GitHub dumps (CERT/CC). While these may provide all required vulnerability information, they are often lacking—the CNVD feed is incomplete as it does not contain changes made to old entries and is only weekly updated with new ones, the CNNVD feed is only available for registered users, without open registration to the service and the CERT/CC feed is updated only once per year. Therefore, these three sources have to be harvested using web scraping, as well as other sources classified in the third category.

Web scraping is performed with custom Python scripts, using the Beautiful Soup [23] library to parse HTML files. A JavaScript engine is needed to retrieve data from the CNVD; therefore, we use a web browser through the Selenium Framework [24] to download it. In other cases Python’s built-in network libraries are sufficient and are used instead. For each source the HTML data is parsed and relevant information is retrieved and stored in a JSON format. Entries in languages other than English are translated using Google Translate API [25]. Since *raw databases* are meant as raw representations of data from the actual remote sources, no other processing is done at this stage. Therefore, the structures of *raw database* entries are vastly different for each source and are incompatible with each other. Additional binary files available from some sources, usually exploit-related, are also downloaded and archived.

Entries stored in *raw databases* are used to create *low databases*, which follow one of the two unified formats, different for vulnerabilities and exploits. Standardizing entry formats allows for easier data aggregation and correlation in subsequent stages. Data processing involved in this step includes:

1. Parsing dates and saving them in ISO 8601 compliant format;
2. Parsing affected products lists to separate vendor, product and version fields;
3. Parsing references to create a list of external IDs, which is used in later stages to correlate entries from different sources;
4. Dividing entries that contain multiple vulnerabilities to create one entry per each vulnerability;
5. Adding IoT classification based on categories, tags, etc. from source.

The process of transforming a *raw database* entry into a *low database* entry is supposed to keep all the data available in the former one; however, some minor losses are possible at this stage. For example, external IDs that do not comply with formats used by their corresponding sources are discarded. The unified low database entry structure for vulnerabilities is presented in Listing A1, found in Appendix A. Each *low database* is synchronized with its *raw database* immediately after any changes to the latter are done, ensuring that data available for the next processing steps is as current as possible.

## 4. Information Aggregation and Correlation

One of the unique features of the VARIoT’s vulnerabilities database is the correlation and aggregation of information about vulnerabilities from different sources. As presented in the previous section, there are a lot of publicly available databases with the information about vulnerabilities in different types of software and hardware. Only a few of these databases are solely dedicated to the IoT or at least somehow indicate such vulnerabilities, but none of them explicitly aggregate information from other sources (beside placing external links—e.g., Vulners.com accessed on 7 May 2021).

**Figure 3:**
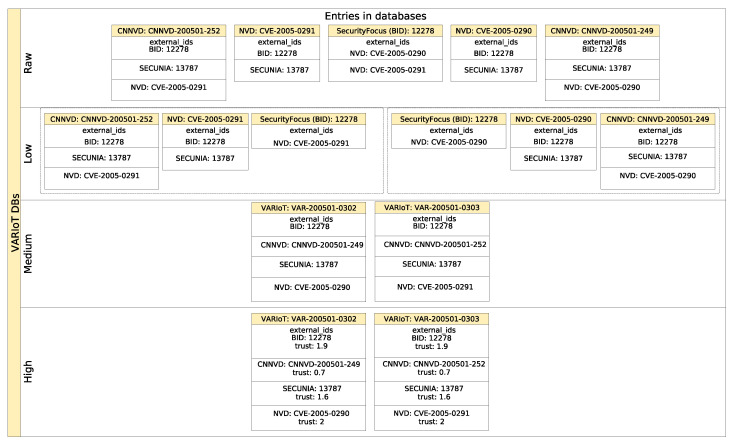
Process of merging entries from *raw databases* into a *high database* entries on the basis of external identifiers.

The aggregation of information about vulnerabilities depends on two key features of data in the *low databases*: the common data format and lists of external identifiers linking vulnerabilities descriptions in different sources. A common data format in the *low databases* helps in integrating data from matching entries into an entry in the *medium database*. External identifiers help to match entries from different databases. To aggregate entries from the *low databases*, at least one external identifier must match and all matching entries (from the *low databases*) must point to the same CVE or not have one. This means that *low databases* entries with matching external identifiers but with different CVEs will not be aggregated. This constraint is mainly intended to limit aggregation. For example, some sources link similar vulnerabilities which are related, for example, by a common software or hardware stack or by the means of exploitation. However, the automatic evaluation of the relation between linked vulnerabilities is very hard to assess (i.e., the scope and severity of linked vulnerabilities may be completely different). Therefore, we decided to limit aggregation to a maximum of one CVE per entry. This process is presented in Figure 3. We have two CNNVD entries, two NVD and one SecurityFocus (BID). These five entries are pointing to two CVEs (CVE-2005-290 and CVE-2005-291). However, only SecurityFocus’s entry points to both. Therefore, it is split into two separate entries in the *low database* (NVD and CNNVD entries are just standardized to a common data format). In the *medium database*, the aforementioned entries from the *low databases* are merged into two entries with different CVE identifiers. In the next step, *medium database* entries are processed into the *high database*.

Every *medium database* entry is a union of data from matching *low databases* entries with explicit information about the source of information in every field. Examples of the aggregation of data are presented in Listing 1, Listing 2 and Listing 3. Listing 1 presents the information from the affected_products fields aggregated for one vulnerability out of three *low databases*: NVD, CNNVD and JVNDB. The affected_products field contains information about the vulnerable software or hardware, including: vendor name, model name, affected versions and scope of affected versions (i.e., equal, newer or older than). Listing 2 presents information from the description field from the three aforementioned databases. This is any text describing nature of vulnerability in more or less detail. In Listing 3 one can find titles aggregated from the *raw databases*. The title field contains a short description of the vulnerability. As one can see, the information is correlated but mostly duplicated, so it would be beneficial to keep only unique ones. The next step after aggregating data is to automatically handle similar data from different sources. Knowledge of the source of the information is needed to estimate how much every part of the data can be trusted. This is helpful in selecting the most reliable and insightful information on vulnerabilities from across all the matching sources.

The process of computing trust and selection of data is part of building the *high database* and is described in the next sections.

**Listing 1:**
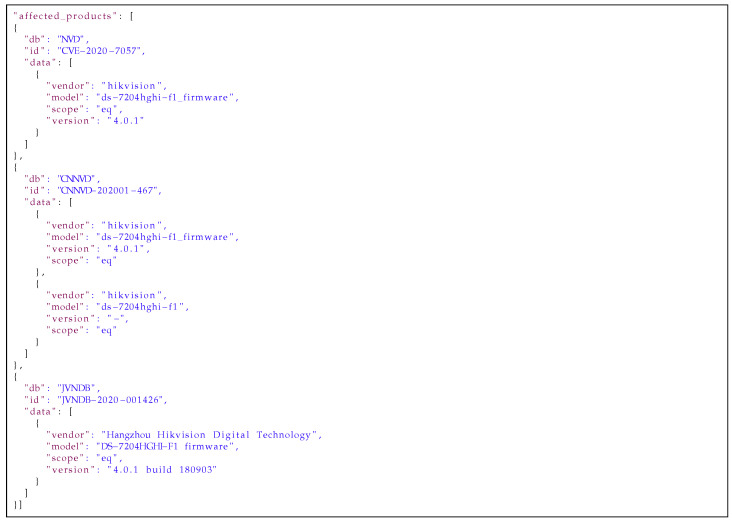
Example of the affected_products field from the *medium database* entry.

**Listing 2:**
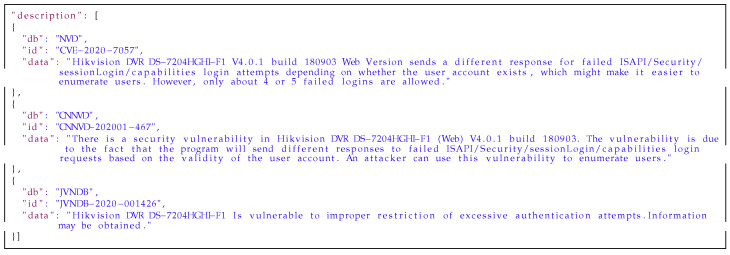
Example of the description field from the *medium database* entry.

**Listing 3:**
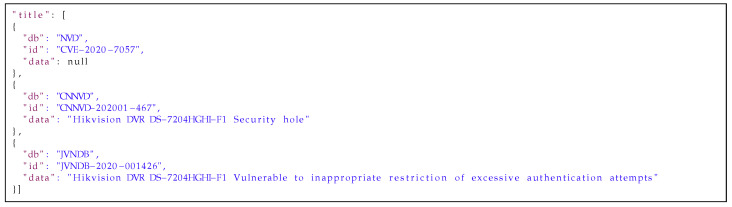
Example of the title field from the *medium database* entry.

## 5. Information Enhancement and Selection

Information in the *high database* should be actionable for people as well as for machines. To achieve this, we need to process different fields of every entry in the *medium database* differently during building the *high database*. For example, the title field is more useful for people. This field can be less structured but should contain insightful yet concise information about the vulnerability. Therefore, for this field, we are selecting only the best and most reliable information. For the description field, we are using a more sophisticated method. To preserve as much information as possible from all the sources, we are using Word Mover’s Distance (WMD) algorithm [26] from Gensim library [27] with the fastText English model [28] to compare sentences of descriptions and get rid of duplicates (sentences from less trusted sources are removed). On the other hand, fields like: affected_products, cpe (affected products’ identifiers in CPE format [29]) or cvss (Common Vulnerability Scoring System as attack’s vectors and single metrics [30,31]) should be easy to use with IT assets management or risk assessment tools. This information must be precise and well structured but it can be broader as it will be processed automatically. For the aforementioned fields, like: affected_products, cpe or cvss we merge the data from all the sources, deduplicate it, sort by the trust level and present as a list of values.

Listing 4, Listing 5 and Listing 6 present results of our algorithms’ work on the data from Listing 1, Listing 2 and Listing 3 on moving an entry from the *medium* to the *high database*. The data in the affected_products field has been deduplicated and sorted by the trust level. The data in the description field has been deduplicated and concatenated on the basis of the WMD algorithm’s results and trust to the sources. Information in the title field has been selected from the most trusted source. All information is presented with sources’ identifiers and trust levels so if necessary, the end users of the database can use their own filtering mechanisms to select data from particular sources or with a particular level of trust.

**Listing 4:**
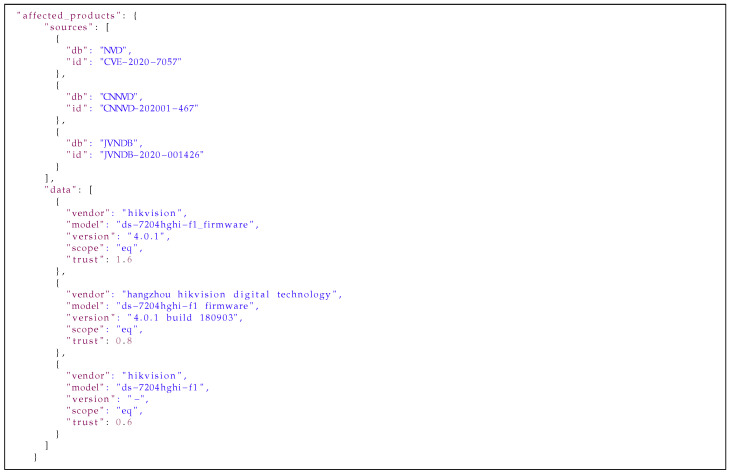
Example of the affected_products field from the *high database* entry.

**Listing 5:**
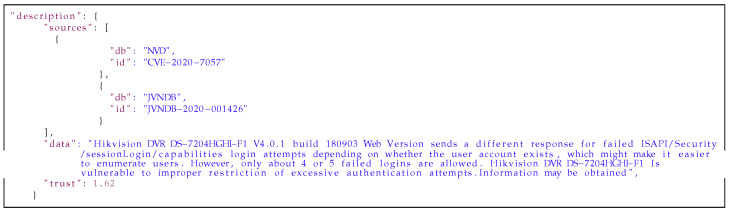
Example of the description field from the *high database* entry.

**Listing 6:**
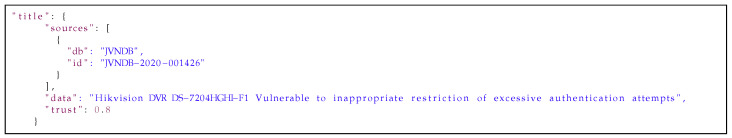
Example of the “title” field from the *high database* entry.

## 6. Metainformation Extraction

The data that we collected in the database was used to create dictionaries with information about vendors, models, device types or vulnerability types and to create a training dataset used to prepare an NLP/AI based solution. The main sources of information for which the mechanism will be used are unstructured data sources such as articles or blog entries about vulnerabilities in IoT devices. The information that can be extracted is as follows. To extract the text keywords from input text we are using the Gensim library [32]. We want to get summaries that will contain about 100 words. If the length of the input text is sufficient to create a summary, we used another method from the Gensim library [33]. Results of these two extraction methods give some general information about text. Information about vendors, device names and device types is based on searching for words or phrases in prepared dictionaries. External database identifiers are extracted with a set of regular expressions collected during other VARIoT works. The metainformation extraction mechanism can also be used to estimate the criticality (CVSS) of the vulnerability presented in the description. We are searching for information about vulnerability types in two ways. The first way is to search for words or phrases from the prepared dictionary. The second method used is the custom Named Entity Recognition (NER) model from the Spacy library [34], based on training data prepared using the VARIoT project’s data. Default NER model implemented in the Spacy library identifies basic information types like organizations, people and dates. We wanted to adapt this model to identify information about vulnerabilities, so it had to be significantly developed and adjusted. The current custom NER model enables identifying the vulnerability types. It can also be used for extracting information about IoT vendors and model names. By using only a dictionary-based solution, we would be putting ourselves at risk of not finding the phrases with different word orders or ways of describing information. With the help of the custom NER model, we can identify information that is not in the dictionary or that we have but in a different form. We used the rule based matching method [35] for preparing the custom NER model training dataset. This Spacy library method allows for the matching of phrases in the input text with previously prepared patterns based on special rules. Patterns take into account the following features of the input text:

- The occurrence of the specific words;
- Part of speech;
- Types of special entity labels generated with the Spacy library;
- Punctuation;
- Case-sensitivity.

The process of creating the NER model is as follows. The first step is creating the training dataset. For each sentence with a phrase about vulnerability type identified with rule-based matching, additional information was added. The first information is an entity label (type of vulnerability). The second information is the string index range of phrase within the sentence related to vulnerability type. A dataset of elements that were prepared in this way was used to train the custom Named Entity Recognition model.

The process of learning the NER model requires training data. The training dataset consists of sentences from the Japanese Vulnerability Database (JVNDB). JVNDB was selected to create the training dataset for two reasons. There are approximately 130,000 entries, which make it possible to create a comprehensive collection of training dataset. The second argument is a regular structure of the presented vulnerability descriptions. Schematic building of JVNDB descriptions allowed automatic extracting information about vulnerability types using rule-based matching.

Below is an example of a description extracted from an article (in the Listing 7) about vulnerability in Cisco devices and results obtained with the metainformation extraction mechanism (in the Listing 8). In this example, the summary and keywords are generated with the Gensim library. In prepared dictionaries were found information about the vendor, product name and device type. The most interesting information which was extracted from the description is about vulnerability type. In both cases: with dictionary and the custom Named Entity Recognition model, vulnerability types were extracted with similar result. The last field in this data are identifiers from external sources mentioned in descriptions.

**Listing 7:**
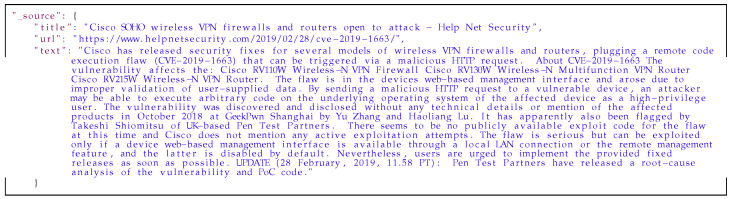
Example of blog entry.

**Listing 8:**
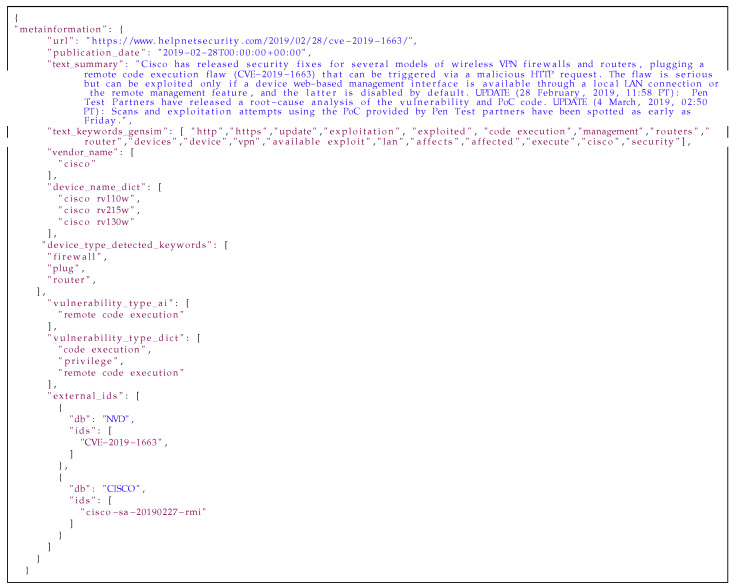
The result of using the metainformation extraction mechanism.

## 7. Trust Management

Trust and reputation management (TRM) is used in various applications such as Wireless Sensor Networks, peer to peer networks, e-commerce platforms and recommendation systems [36,37]. It can be used to evaluate trust of vendors of IT products [6] or IoT devices but also for information processing methods. In our work, trust estimation is used to select the most reliable and informative piece of information as well as to evaluate its reliability.

For each source, its trust value (source trust—$T_{S_{k}}\in \left[0,1\right]$) was assigned on the basis of expert’s knowledge, taking into account properties such as reliability, accuracy and comprehensiveness of information presented in the source, recognition of the source in the community, documentation or available information related to the source, stability and topicality of the source, uniqueness of information provided, self-consistency of information within the source and also consistency with information from other sources (by providing links to other sources or identifiers of entries in other sources). Source trust values for each source are presented in Table 2.

For most of the fields, the same values of each field are aggregated and sum of trust values of sources presenting the same information are calculated. That action is performed for each existing value of that field (for each piece of information). Let us assume that sources *k* till *m* have the same information (information—*i* related to a field *f*). Then, the trust for that piece of information can be calculated as follows:

(1) $$T_{f_{i}}=\sum_{j=k}^{m}T_{S_{j}}$$

After calculation of the trust value for all pieces of information, the piece of information which has the higher trust value is selected as the most reliable.

As we have indicated in the previous sections, we use another method to create a description which will contain and aggregate all information form all descriptions related to a particular vulnerability. However, alternatively we can also use trust to select a description from the set of existing descriptions. To calculate trust to piece of information related to description field, modified method of trust calculation should be used. However, every description is rather unique (if not, it means that one source simply copied the description from another, because the probability of independent repetition of long text is rather small), we have assumed that even if a source has copied a description from another source (these two sources are not independent), we hope that some of the sources also do a simple verification of that description, so if the same description is present in more than one source we can trust that description more than the description provided only by one source. On the other hand, we want to take into account the length of the description. Because of that we calculate trust to specific description by taking into account the source trust of sources which had provided such description and the length of the description (we assume that the longer the description, the better, as it could be more informative). So to calculate trust to a description *d* (provided by sources *k* till *m*), we use the following formula:

(2) $$T_{desc_{k}}=\sum_{j=k}^{m}T_{S_{j}}n_{d}$$

where $n_{d}$ is the length of description *d*.

To some extent a source can present more reliable information regarding a field but less reliable information in the case of other fields. Because of that, a more advanced approach could be implemented, in which source trust value would be set not only in relation to every source but to every field in every source. It would lead, however, to a significant increase in complexity of trust mechanism and multiplication of source trust values which should be taken into account in the mechanism. However, the necessity of evaluation of a few times more trust values is the worst feature connected with that advanced mechanism and it would be hard to be done on the basis of experts method, which is the most reliable in that case. Moreover, such advanced trust mechanism probably would not introduce significant changes in the trust results in the case of a vast majority of evaluated pieces of information.

It is worth emphasizing that trust management is used to select the most reliable information, when many pieces of information (dissimilar) from various sources exist but also for an estimation of how much we can rely on the selected information. In theory, to accomplish these two aims (at least to some extent), other means could be used, such as natural language processing or machine learning. NLP could be used to try to match information obtained from various fields (for example between description and cvss) in a way that we use it to extract such information from description in case it is not provided separately, as explained in the previous section. It is less useful, however, in the case when inconsistent information from various sources exists. On the other hand, we could try to use other machine learning methods, but the accurateness of the selection process would be in that case hard to evaluate and the process of selection would also be hard to digest, analyze, audit and improve. Because of this we decided to use the natural and easy to understand concept of trust. The concept of trust also has more benefits than simple prioritization of sources because it takes into account the very typical situation when many sources present consistent information within a field.

As indicated earlier, in case the same information exists in more than one source, we assume that this information is more reliable (we sum up trust values connected to all sources that provide such information). That way of operation is valid when we are certain that all sources are independent from each other. However, it is not always true in relation to sources of information about vulnerabilities and exploits—we have observed that a few sources duplicate information harvested from other sources without further verification. We have taken into account such situation by adjusting (namely: lowering) the source trust value of sources which copy information from other sources.

## 8. Results

**Figure 4:**
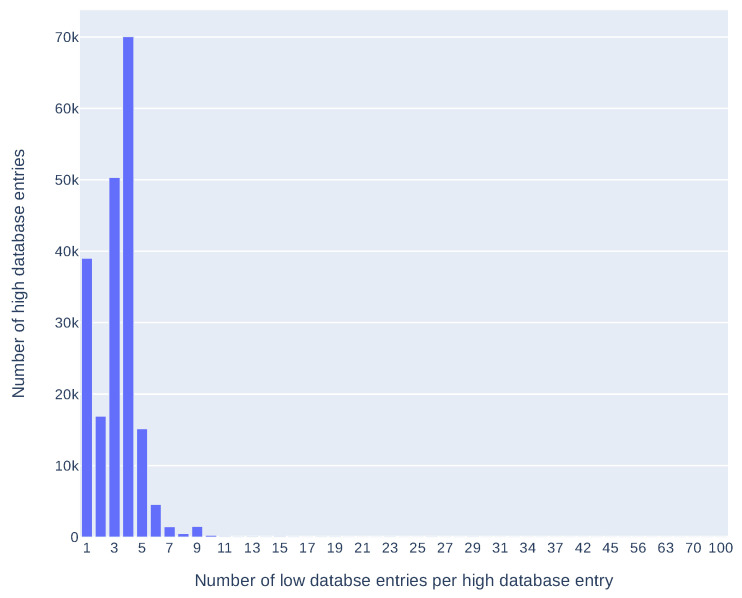
Number of *high database* entries containing data from a given number of the *low database* entries.

There are 650,494 entries in *low databases* which gives 203,475 entries in the *medium* and the *high database*, where 19,572 are IoT related (as of 21 May 2021). About 80% of all entries in the *high database* contain data from more than one source (mostly from two to four). Detailed results can be found in the plot in Figure 4.

**Figure 5:**
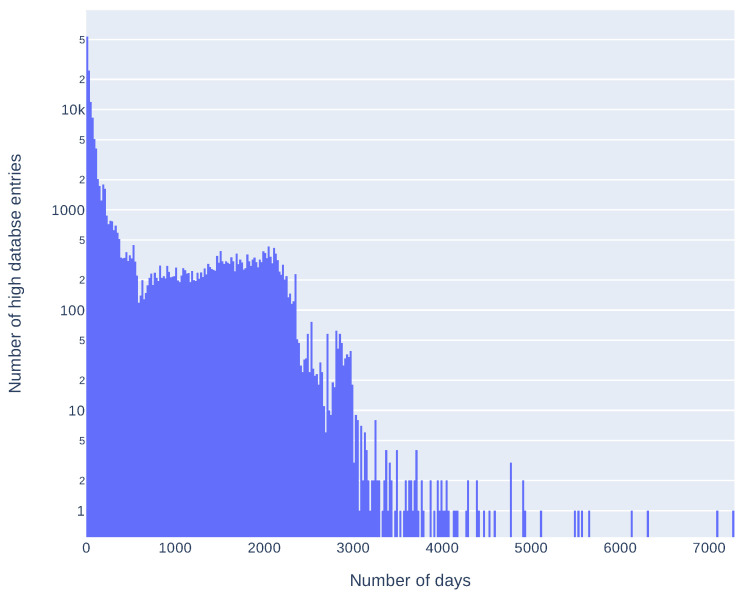
Number of entries in the *high database* with given delay (in days) between first and last source publishing information about vulnerability (Y axis is logarithmic).

**Figure 6:**
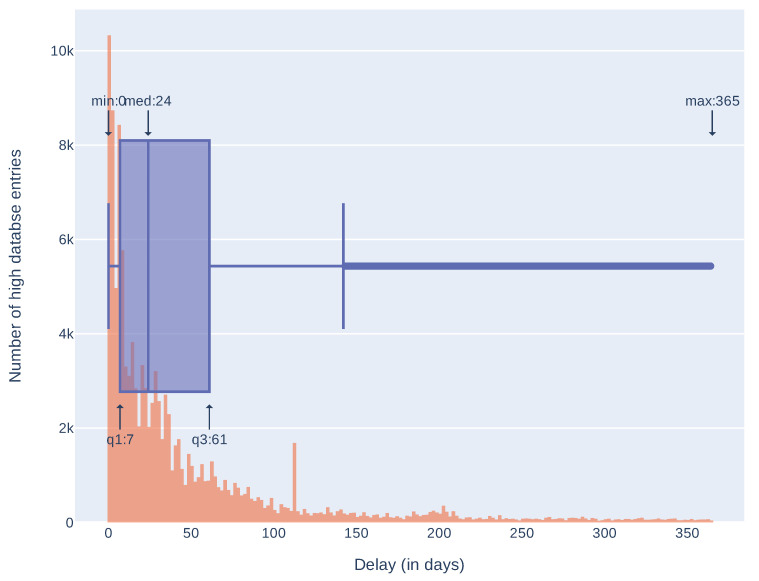
Number of entries in the *high database* with given delay (shorter than 365 days) between first and last source publishing information about vulnerability presented as a histogram and a box plot. Highlighted are: minimum = 0 days, maximum = 365, median = 24 and quartiles 1 and 3 (7 and 61 days respectively).

**Figure 7:**
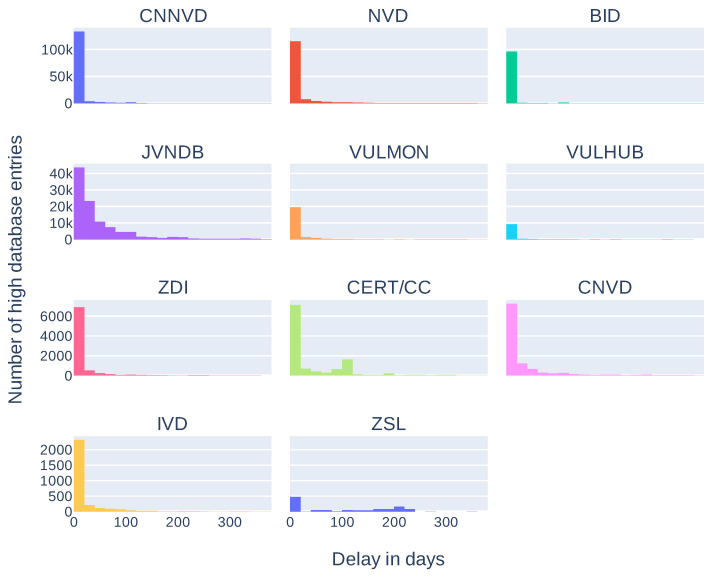
Histograms of number of entries in the *high database* with given delay (in days, since first publish date) in publishing vulnerability information per source.

**Figure 8:**
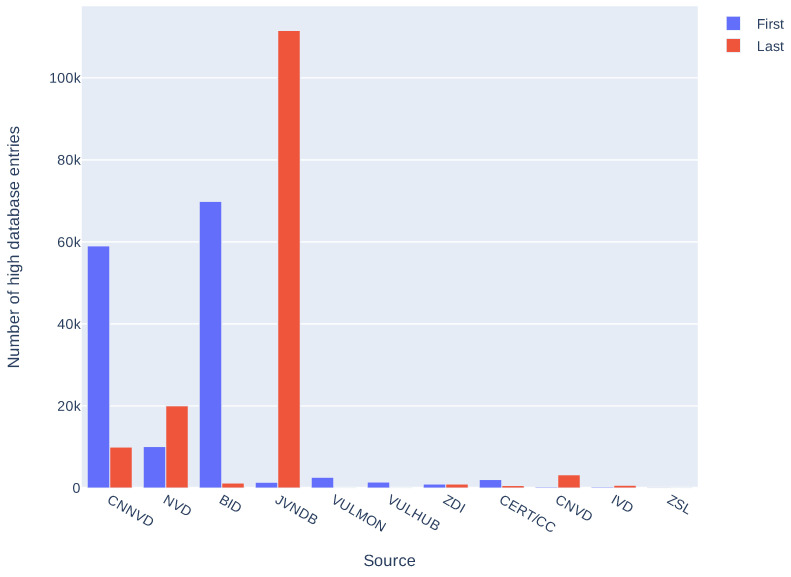
Number of entries in the *high database* where given database reported vulnerability as a first one or a last one.

Additionally, we compared release dates of information in sources composing the *high database* entries to see how many days can pass between the first and the last source publication. Results are presented in Table 3 and in Figure 5. These results take into account only *high database* entries with more than one source and more than one correct release date (146,484 entries). As some of the sources place invalid release dates (for example, beginning of the Linux epoch, i.e., 1 January 1970), we arbitrarily took into account only the dates after 1 January 2000. It is not a complete solution, for example, CNNVD has a history of faking release dates [38] but removes obvious errors. Seventy five percent of entries have a difference less than 166 days. Differences over 2000 days (more than five years) are mostly an effect of wrong release dates presented in the sources (as described above). As most of the differences are less than a year we also analyzed delays in this time span. Results, presented in Figure 6, show that most of the differences between the first and the last mention in the sources are less then 61 days. Histograms of delays (in days) for every source are presented in Figure 7 and statistics in Table 4. We have also checked which databases most frequently report vulnerability as first and which as a last one. The three databases that report the most frequently as the first one are: SecurityFocus (BID), CNNVD, and NVD. The three most frequently reporting as last one are: JVNDB, NVD, and CNNVD. NVD and CNNVD are in both top threes because these are also some of the biggest databases, therefore raw counts (not normalized) outnumber other, smaller databases. Results for all the sources are presented in the Figure 8.

Merging information from many sources enhances vulnerabilities’ data completeness. To illustrate benefits of this process we present an example of the entry from the medium database in Listing A2 in Appendix B. Even though this vulnerability has no CVE identifier, the CNVD database provides the CVSS vector so one can assess the risk related to this vulnerability (the cvss field). On the other hand, Zero Science Lab (ZSL) provides a lot of external links for further reading or even with an exploit (the references field). Finally, ZSL and CNVD present slightly different details of the affected products (the affected_products field). However, this form of data is not optimal as it contains duplicated or missing information. To make it more useful, the data is deduplicated, aggregated and selected as described in Section 5. The results of these operations are presented in Listing A3 in Appendix B containing an example of the high database entry corresponding to the same vulnerability. To prove the validity of the method it is worth analyzing the level of completeness of information achieved in the *high database*. To do this we want to show statistics regarding the lack of information in particular fields. It is worth emphasizing that these statistics are done before our extraction mechanism has operated. Table 5 summarizes achieved statistics about information completeness. Of course, on that stage we do not try to analyze accurateness of information, just the completeness of data. We will try to evaluate accurateness of information by taking into account trust management results at the end of this section.

As can be seen from Table 5, in relation to entries about IoT vulnerabilities, information regarding vulnerability description, affected products or vulnerability risk assessment is quite complete (higher than 99%). In relation to all entries (also regarding IoT), the completeness of information is lower. To achieve even better results of data completeness or handle unstructured data sources (e.g., blog posts or news) in the future, we have developed an NLP/AI tool to extract metainformation from a text as described in Section 6. It is due to the fact that description of vulnerability is the most common type of information both in structured (as can be seen in Table 5 in relation to sources harvested by us) and unstructured sources (as then it is the only type of information which can be harvested directly).

Below we present the evaluation of the metainformation extraction mechanism. For the purpose of analyzing the results generated by the mechanism, a test dataset was prepared with high database entries tagged as IoT information with descriptions and defined vulnerability types. Taking into account the criteria presented above, it was possible to obtain 14,841 entries from the *high database*. The next step was using the mechanism on the test dataset and providing quantitative data of metainformation extraction results. General statistics can be found in Table 6.

Text keywords (“text keyword gensim”) were generated for all entries and summaries (“text summary gensim”) in 99% of entries. In 56% of entries information about vulnerability type with the custom NER model (“vulnerability type ai”) was found, but with vulnerability type dictionary (“vulnerability type dict”) this information was found in 95% of entries. Metainformation extraction process was able to obtain information about vendors (“vendor name”) from 82% of entries and devices (“device name”) from 61% of entries.

For a deeper analysis, we conducted a verification of the results of the metainformation extraction process’s results. We have chosen vulnerability type as this type of information has the smallest level of completeness in relation to IoT vulnerabilities, as can be seen in Table 5. We made this verification by comparing the types of vulnerabilities generated by the mechanism with the information contained in the test dataset (the CWE dictionary identifier or a phrase defining the vulnerability type).

**Figure 9:**
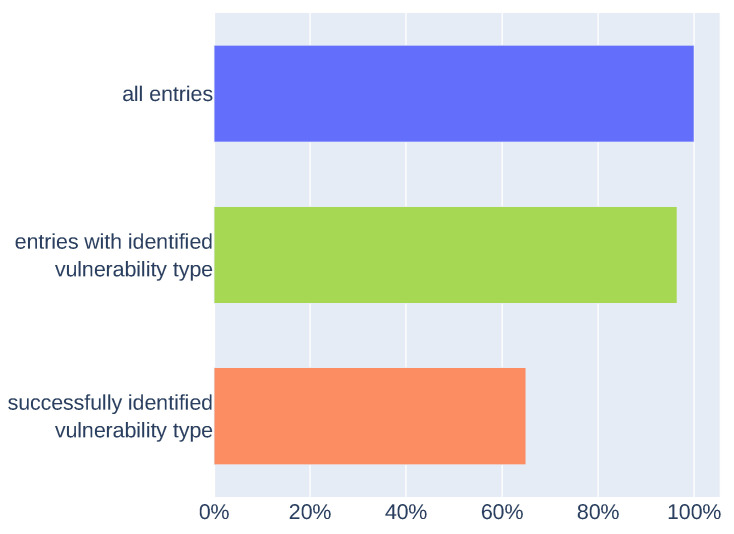
Comparing extracted vulnerability types with vulnerability type information from the test dataset.

Results (presented in Figure 9) show the comparison of extracted vulnerability types (with the custom NER model or vulnerability type dictionary) with vulnerability type information from test dataset entries. Data defined as “all entries” are the whole test dataset. The mechanism was able to extract information about vulnerability type using the custom NER model or dictionary from 96% of entries (“entries with identified vulnerability type”). For 65% of entries, extracted vulnerability types matched the vulnerability type information assigned to specific entry (“successfully identified vulnerability type”). The result obtained after verifying the metainformation extraction may be higher. This may be due to false negatives in the results, which may be caused by how the descriptions in the CWE dictionary or phrases that define the vulnerability type are structured.

**Figure 10:**
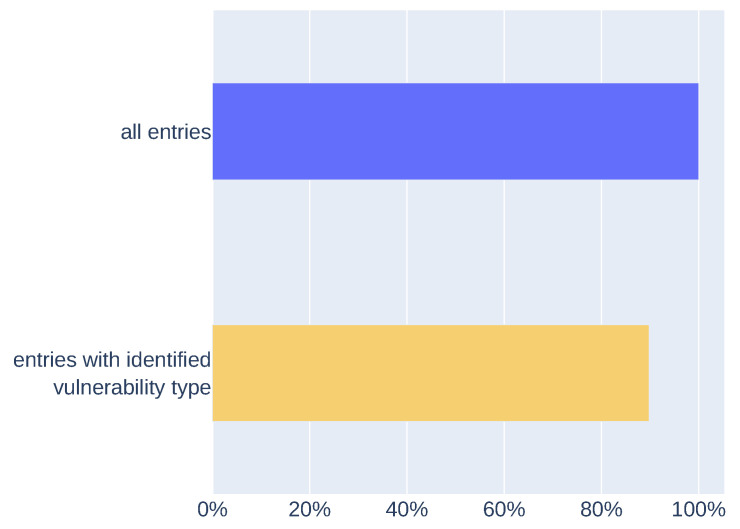
Results of vulnerability type metainformation extraction on data without information about vulnerability type.

In addition to the metainformation extraction performed on the test dataset, we made the extraction process on the other data. In Figure 10 we present results of metainformation extraction on high database entries without information about vulnerability type. There are 4731 entries that are tagged as information about IoT devices and have not defined any information about vulnerability type. We extracted vulnerability types (with custom NER model or with the dictionary) for 89% of entries (“entries with identified vulnerability type” in Figure 10). The results are similar to those presented in Figure 9 (“entries with identified vulnerability type”). In this case, we cannot verify results because of a lack of information about vulnerability type, but quantitative results are similar to those generated on the test dataset.

On the level of the *high database* we have faced two types of problems. The first one, namely: the lack of information in relation to a specific field, we tried to solve by using our extraction mechanism and the results are presented above. The second problem is related to existence of various information related to a field which can be mutually exclusive. For example, a vulnerability can have CVSS score set as 5.0 in one database and as 10.0 in another. To solve that problem we use our trust mechanism as described earlier. In the next paragraphs we want to show some statistics related to the effectiveness of our mechanism to select the most reliable information.

First of all we want to calculate the average trust value within all fields (of course we have taken into account only the highest value of trust related to that field within a vulnerability—we analyze just the value of trust of the most trusted information). We also show the minimum and the maximum of the highest trust value. It is worth noting that trust level above 1.0 implies that the information which is under trust assessment was presented by more than one source, whereas trust level equal or lower than 1.0 does not imply that the information was presented only by one source. The results also show information as to which field is the most often repeated among many sources. The results are shown in Table 7.

The results show that, on average, information in fields related to vulnerability risk assessment, vulnerability type or affected products, is aggregated from more than one source (it is indicated by average trust value to these types of information significantly higher than 1.0). Such information is often repeated (or set independently) by various sources and because of that these fields are very susceptible to the aggregation process. Of course fields such as title are almost always unique, so they have the lowest average trust value. On the other hand, description field has the highest average trust value, despite the fact it is unique, but that is due to the another trust calculation formula.

The only field which has a numerical value and which can be put under more in-depth analysis is CVSS score (vulnerability risk assessment). In all IoT-related vulnerabilities we have analyzed the dispersion of the value of CVSSv2, and the results can be found in Table 8. We take into account the highest difference within an entry, both in relation to CVSSv2 score and trust value related to that score.

Results presented in Table 8 show that the dispersion of risk assessment for an entry could be very high (max = 10, which means that one source indicates that a vulnerability has CVSS score equal to 0—the lowest possible value but another that this vulnerability has CVSS score equal to 10.0—the highest possible value), but the average CVSS difference is rather small. This shows that sources are rather compliant in relation to risk assessment. As can be seen, the difference between trust values related to that information is rather small on average.

The results regarding trust evaluation show that trust mechanism works as intended and can be used as an evaluation of accurateness of provided information. All results show that the created database of vulnerabilities and exploits could be beneficial and useful to the community of IoT cybersecurity analysts, as it is as comprehensive as possible on the basis of public sources of such information.

## 9. Summary and Future Works

This article has shown that the process of gathering and automatically processing actionable information on IoT vulnerabilities in order to obtain the best results is a nontrivial task, but it is currently necessary to perform it. Obtaining information on vulnerabilities will make it much easier to manage the security of IoT devices. Due to the very rapid growth in the use of IoT devices, they are more and more often used in attacks, and the lack of security measures may lead to the fact that attacks will become more and more frequent. To avoid this, it would be advisable to make users or network owners aware of the vulnerabilities in these devices. Until now, information about them could be found in various places; they were fragmented, incomplete and often unstructured. Creating a publicly available structured database of information about known technical vulnerabilities and exploits is of great benefit to all interested parties: users and producers or network owners.

Our research has shown that by collecting data from various sources, we can obtain a more comprehensive entry than from a single source. Due to the fact that our database collects, correlates and aggregates data from various sources, each entry is rich in actionable information and it also reduces the risk of lack of data or delays in obtaining information on vulnerabilities.

As future works we will enhance our metainformation extraction mechanisms to support other types of information and also we want to further evaluate that mechanism. We will also harvest information from other (also unstructured) sources, which will significantly increase usefulness and also necessity to use our metainformation extraction mechanism. We also plan to create a search engine optimized to find information on the Internet related to the IoT vulnerabilities and exploits.

The most important implication of our research is the fact that there is still much work to do to improve vulnerability management regarding IoT. To move towards that goal, we have focused on providing more comprehensive and reliable actionable information. Mechanisms implemented and information provided by our work can be a ground for building various services. The database could be used by vulnerability scanners—not as an engine of scanning process but as a repository of information about vulnerabilities. That is due to the fact that information collected by us is to some extent broader and more ample than information used by common vulnerability scanners in the context of IoT. Another natural and easy to build but still very practical service could provide a list of possible vulnerabilities on the basis of the product name (vendor and model). Of course to do this the IoT asset inventory must be done beforehand, but such approach can give much better results that scanning (for example, due to the fact that scanners can improperly recognize a device and still not verify the real existence of a vulnerability).

The article is written on the basis of the results obtained during the work in the Vulnerability and Attack Repository for IoT project [39]. This project involves not only creating and sharing information about vulnerabilities and exploits but also scanning the Internet in order to obtain a security image in IoT devices. Laboratories to test legitimate and malicious IoT traffic, IoT artefacts and IoT anomaly models were also built. Moreover, various types of statistics related to devices in a given country will be created. All these tasks are performed in cooperation with our partners, i.e., Stichting The Shadowserver Foundation Europe, Security Made In Letzebuerg G.I.E., Institut Mines-Télécom and Mondragon Goi Eskola Politeknikoa Jose Maria Arizmendiarrieta S COOP.

Data prepared by us will be available on the European Data Portal (through National Data Portals, including the Poland’s Open Data Portal [40]), as well as on many other sources such as MISP platform (Malware Information Sharing Platform), which is commonly used by the community of cybersecurity analysts.

## Figures and Tables

**Table 1:** Summary of used information sources.

Ref.	Short Name	Full Name	Type
[8]	Packet Storm	Packet Storm Security	Vulnerability/Exploit
[9]	Exploit-DB	Exploit Database by Offensive Security	Exploit
[10]	NVD	National Vulnerability Database	Vulnerability
[11]	CNVD	China National Vulnerability Database	Vulnerability
[12]	CNNVD	Chinese National Vulnerability Database of Information Security	Vulnerability
[13]	IVD	ICS Vulnerability Database	Vulnerability
[14]	BID	SecutiryFocus Bugtraq	Vulnerability/Exploit
[15]	JVNDB	Japan Vulnerabilities Notes Database	Vulnerability
[16]	CERT/CC	Carnegie Mellon University CERT Coordination Center	Vulnerability
[17]	VUL-HUB	VUL-HUB Information Security Vulnerability Portal	Vulnerability
[18]	Vulmon	Vulmon Vulnerability Search Engine	Vulnerability
[19]	ZDI	Zero Day Initiative	Vulnerability
[20]	ZSL	Zero Science Lab	Vulnerability

**Table 2:** Source trust of all sources.

Source Name	Source Trust–$T_{S}$
Packet Storm	0.1
Exploit-DB	0.9
NVD	1.0
CNVD	0.6
CNNVD	0.6
IVD	0.2
BID	0.3
JVNDB	0.8
CERT/CC	0.8
VUL-HUB	0.1
Vulmon	0.1
ZDI	0.7
ZSL	0.1

**Table 3:** Statistics of differences in release dates..

Stat	Number of Days
mean	311
std	613
min	0
25%	9
50%	36
75%	166
max	7085

**Table 4:** Release dates delays statistics for sources. Mean, standard deviation (std), minimum (min), maximum (max) and percentiles (25%, 50%, 75%) are in days.

Source	Considered Entries	Mean	std	Min	25%	50%	75%	Max
BID	101,424	6	30	0	0	0	0	365
CNNVD	147,044	11	37	0	0	1	5	365
NVD	142,880	23	56	0	0	1	10	364
JVNDB	104,411	48	63	0	8	26	62	365
Vulmon	23,719	19	51	0	0	1	9	365
VUL-HUB	11,045	19	54	0	0	1	7	364
ZDI	8215	14	40	0	0	1	7	350
CERT/CC	11,643	41	63	0	0	4	84	365
CNVD	10,714	32	60	0	3	8	30	364
IVD	2989	22	48	0	2	5	15	365
ZSL	1126	94	92	0	0	73	193	357

**Table 5:** Completeness of information in the *high database*.

Information	Fields	All Entries	IoT Related Entries
Vulnerability description	description	191,077 (94%)	19,572 (100%)
Affected products	affected_products	154,814 (76%)	19,285 (99%)
Vulnerability type or nature	problemtype_data, threat_type	176,400 (87%)	14,841 (76%)
Vulnerability risk assessment	cvss.data.severity, cvss.data.cvssV2, cvss.data.cvssV3	162,054 (80%)	19,572 (100%)
IoT taxonomy (category or subcategory)	iot_taxonomy	-	12,677 (65%)

**Table 6:** Metainformation extraction results.

Metainformation Extraction Parameter	Success Rate
all entries	100%
text keyword gensim	100%
text summary gensim	99%
vulnerability type dict	95%
vulnerability type ai	56%
vendor name	82%
device name	61%

**Table 7:** Statistics of the highest trust value within the most important fields.

Information	Min	Average	Max
Vulnerability risk assessment	0.2	1.50	5.6
Vulnerability type or nature	0.8	1.57	3.5
Affected products	0.2	1.20	5.6
Description	0.2	1.98	6.12
Title	0.2	0.81	3.5

**Table 8:** Analysis of dispersion of CVSSv2 (risk assessment) and trust value related to CVSS.

Value	Min	Average	Max	Standard Deviation
cvss difference	0.0	0.43	10.0	1.03
difference of trust value of cvss	0.0	0.28	5.4	0.59

**Listing A1:**
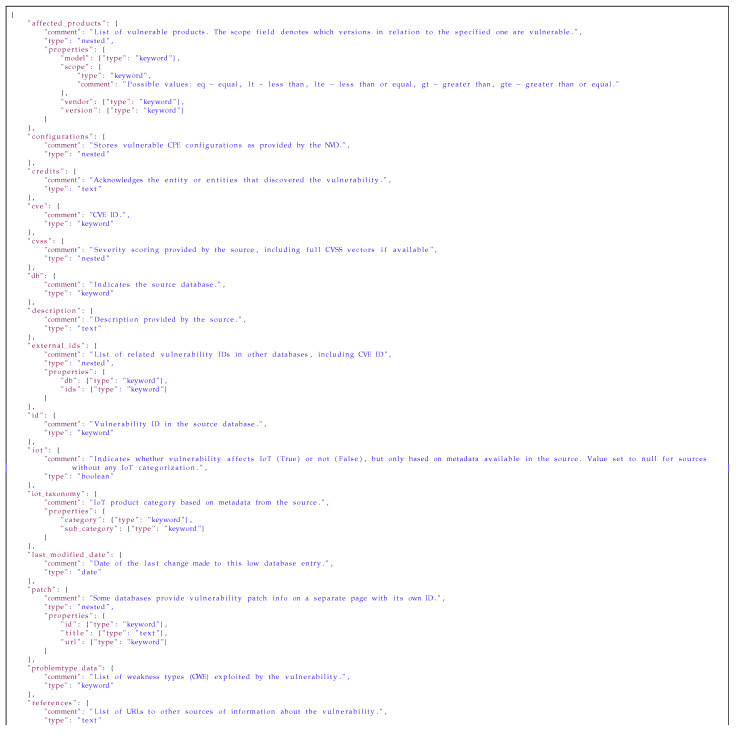

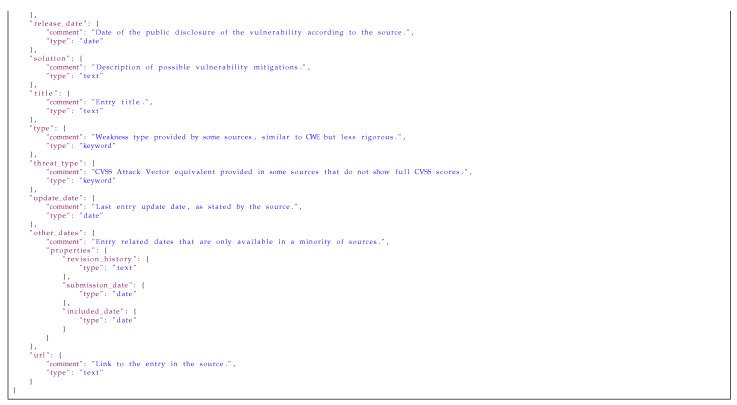
Common low database data format.

**Listing A2:**
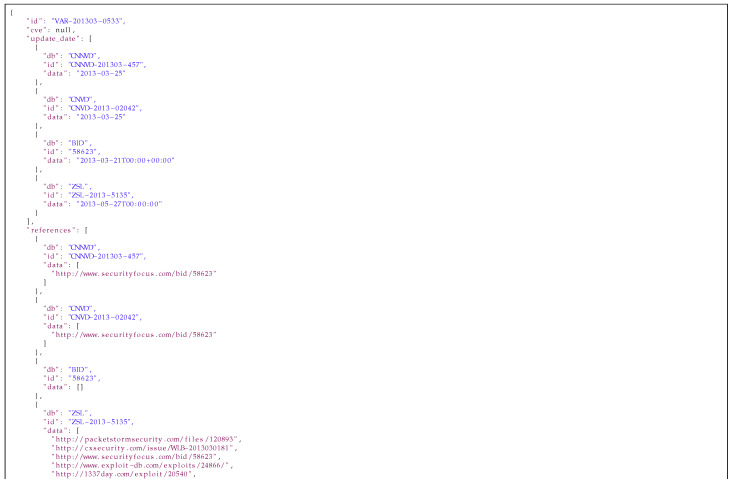

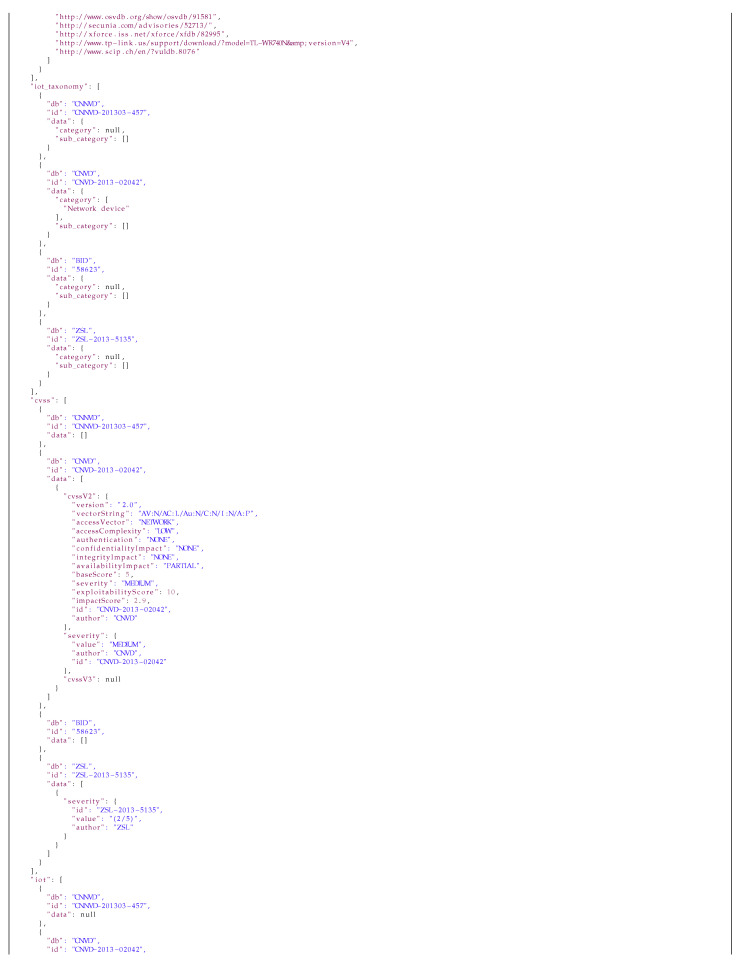

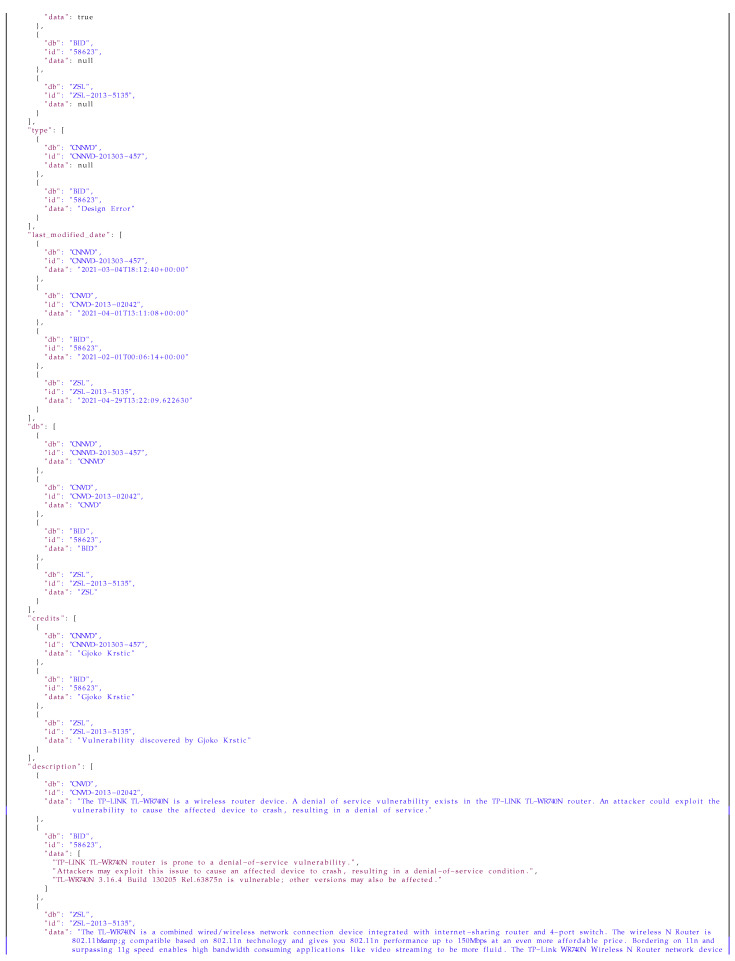

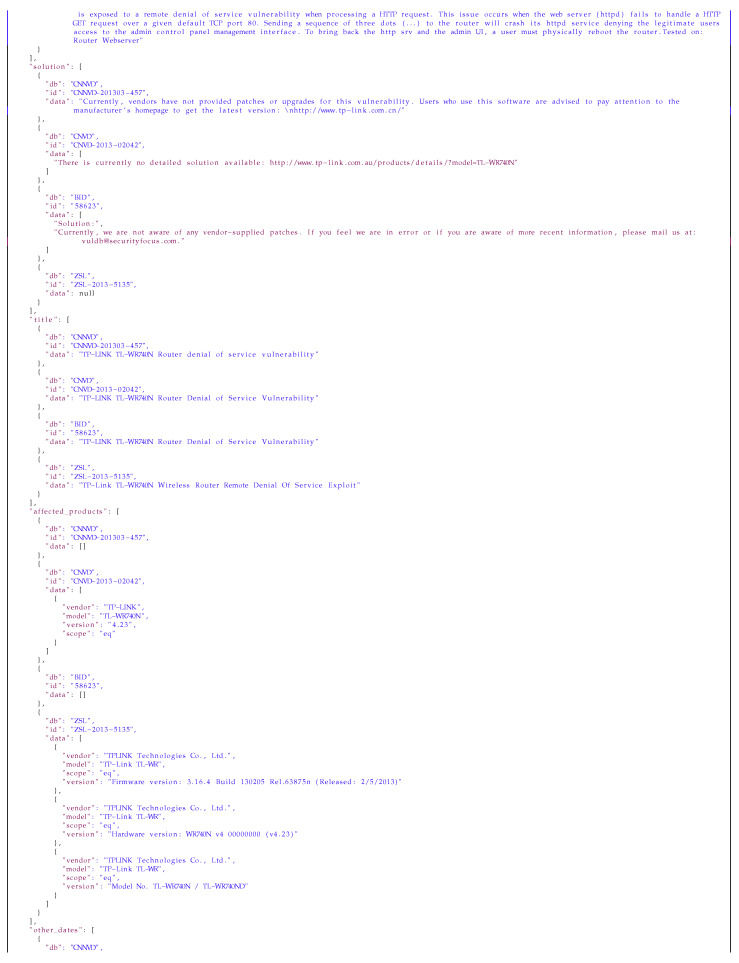

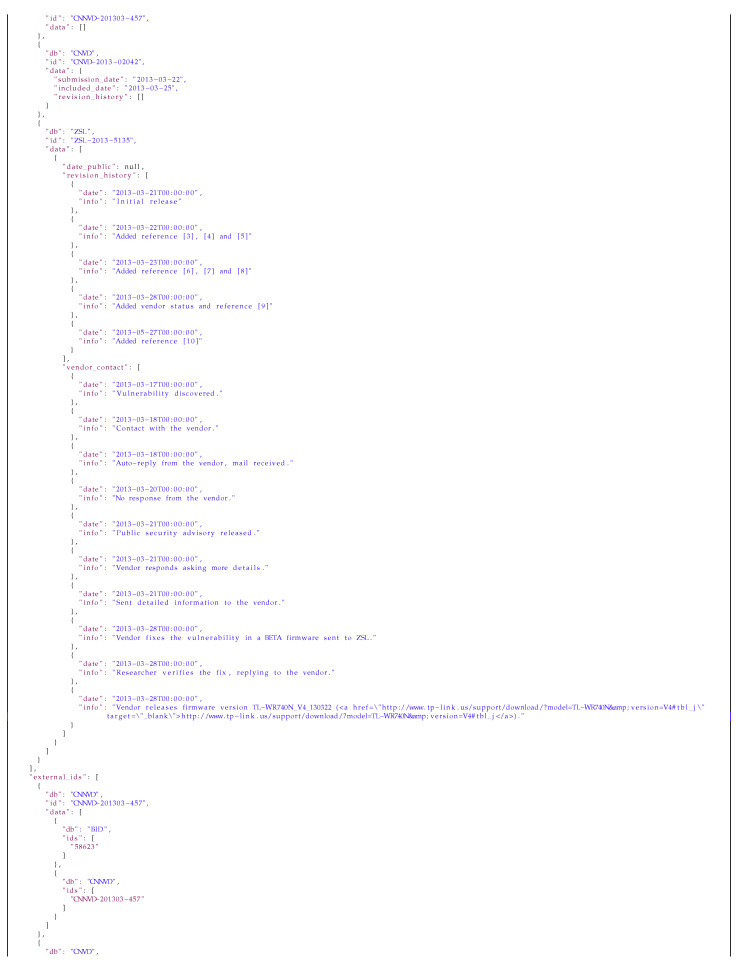

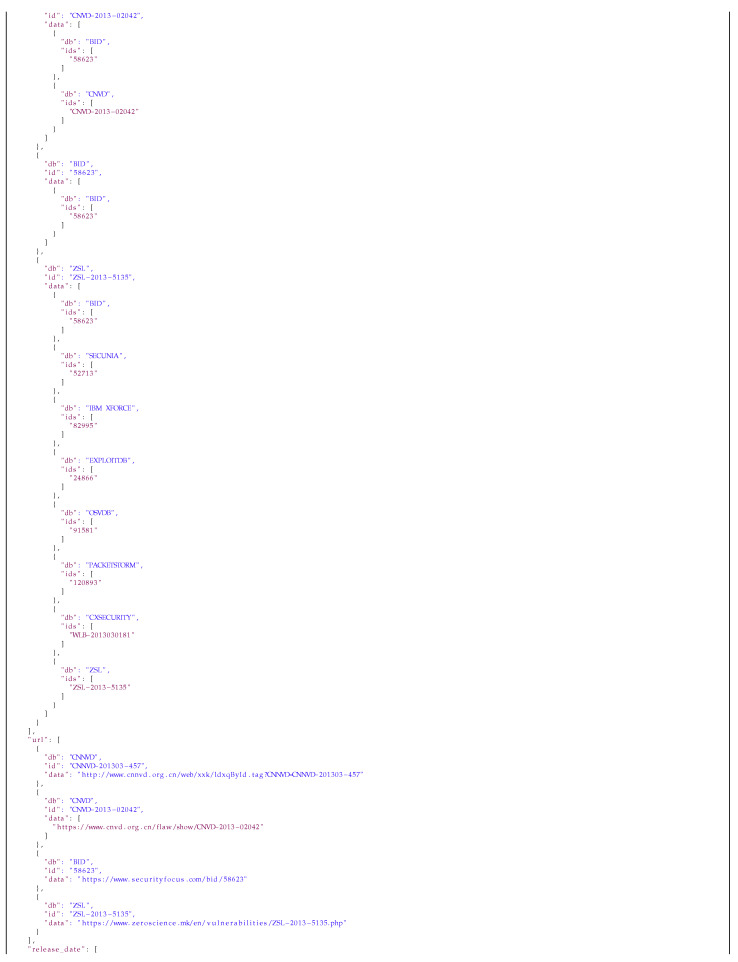

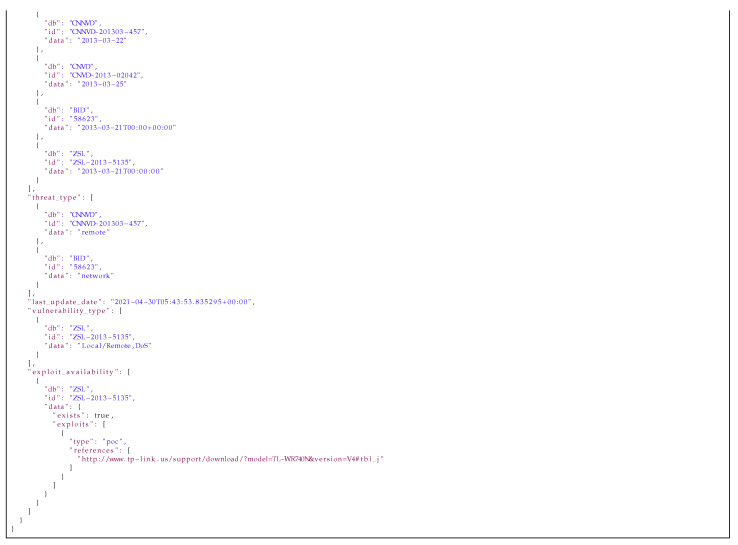
Example of the entry from the medium database.

**Listing A3:**
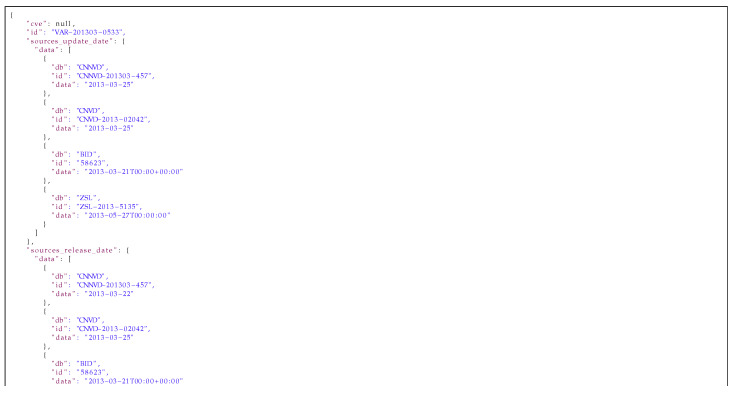

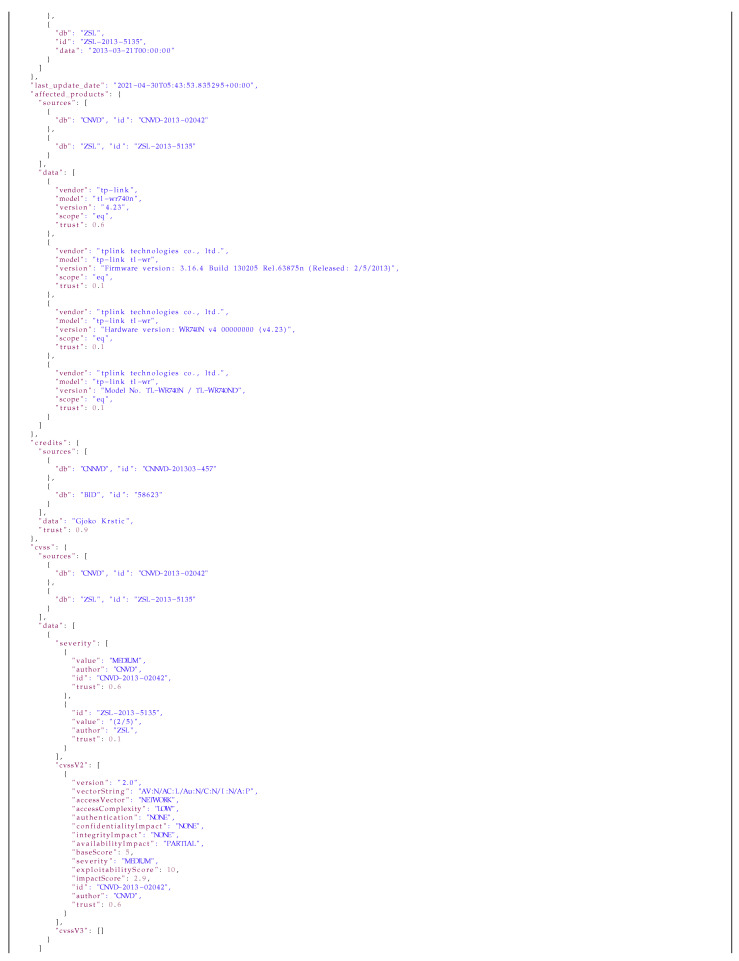

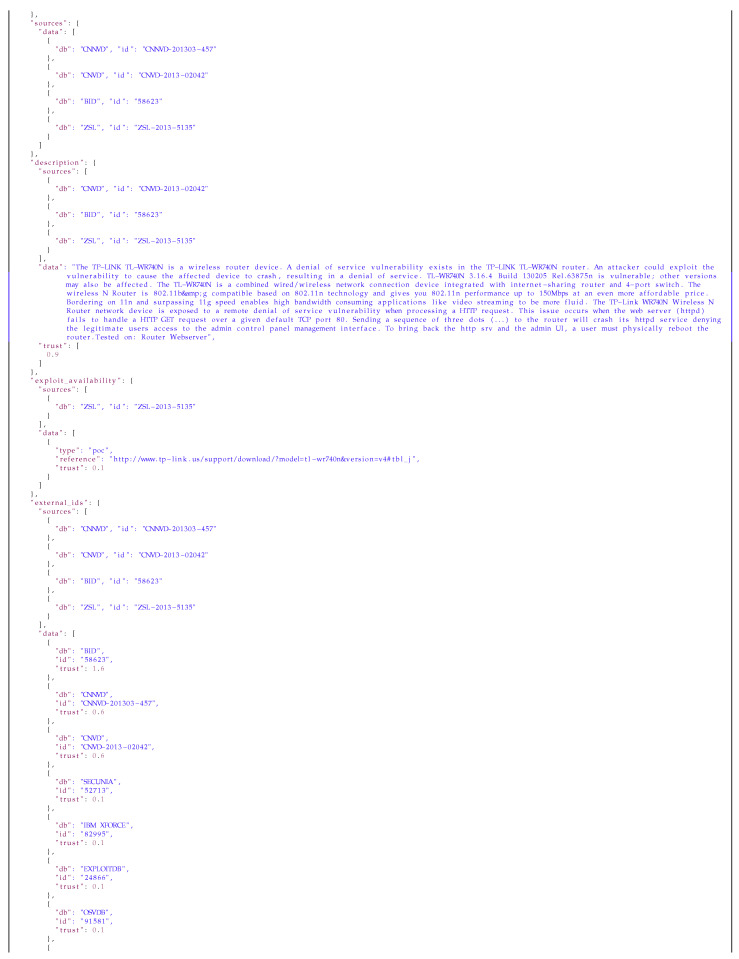

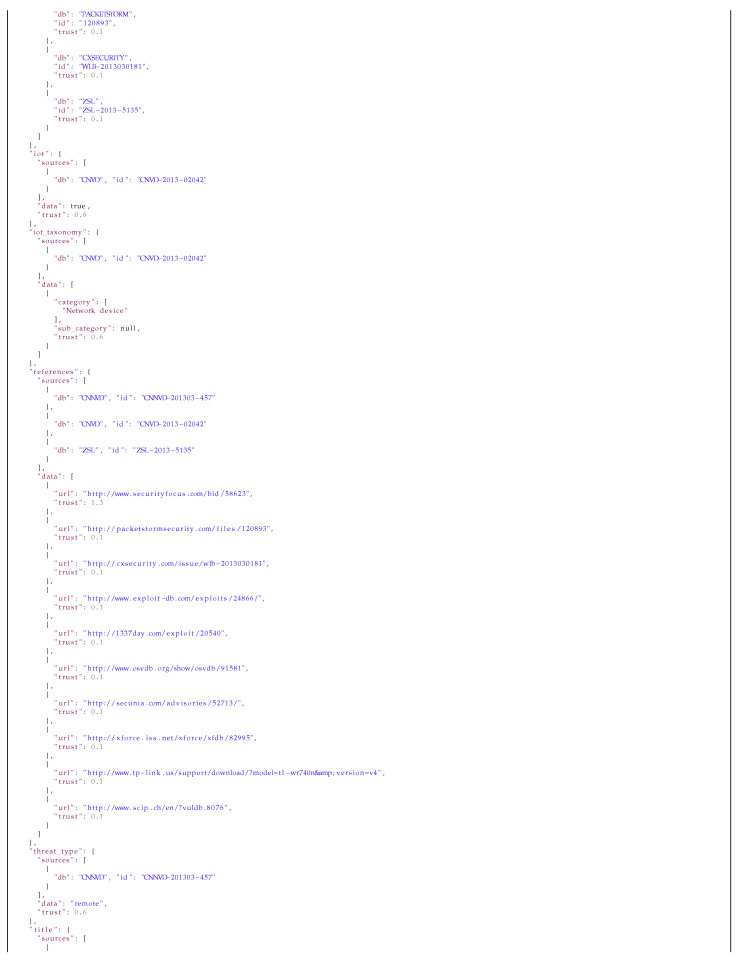

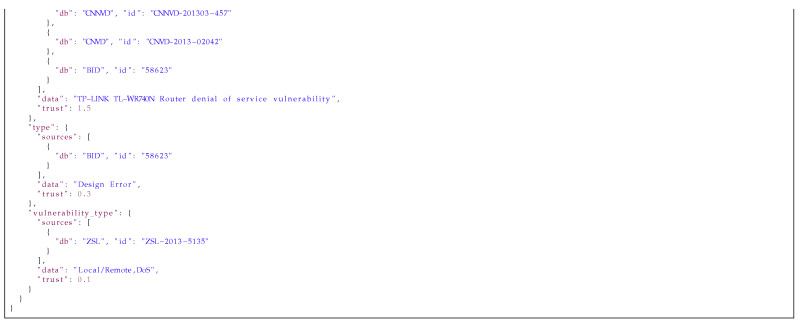
Example of the entry from the high database.

## Data Availability

Data will be available through European Data Portal by the end of 2021.

## Abbreviations

The following abbreviations are used in this manuscript:

IoT	Internet of Things
TRM	Trust and Reputation Management
AI	Artificial Intelligence
NLP	Natural Language Processing
CVSS	Common Vulnerability Scoring System
CWE	Common Weakness Enumeration
CVE	Common Vulnerabilities and Exposures
